# Nasal turbinate lymphatic obstruction: a proposed new paradigm in the etiology of essential hypertension

**DOI:** 10.3389/fmed.2024.1380632

**Published:** 2024-08-16

**Authors:** William Thomas Phillips, Joyce Gensberg Schwartz

**Affiliations:** ^1^Department of Radiology, UT-Health San Antonio, San Antonio, TX, United States; ^2^Department of Pathology, Methodist Hospital, San Antonio, TX, United States

**Keywords:** hypertension, intracranial pressure, Cushing’s mechanism, glymphatics, sympathetic activity, parasympathetic activity, brain self-protection, brain blood flow resistance

## Abstract

Hypertension affects an estimated 1.3 billion people worldwide and is considered the number one contributor to mortality via stroke, heart failure, renal failure, and dementia. Although the physiologic mechanisms leading to the development of essential hypertension are poorly understood, the regulation of cerebral perfusion has been proposed as a primary cause. This article proposes a novel etiology for essential hypertension. Our hypothesis developed from a review of nuclear medicine scans, where the authors observed a significantly abnormal increase in nasal turbinate vasodilation in hypertensive patients using quantitative region of interest analysis. The authors propose that nasal turbinate vasodilation and resultant blood pooling obstruct the flow of cerebrospinal fluid passing through nasal turbinate lymphatics, thereby increasing intracranial pressure. The authors discuss the glymphatic/lymphatic clearance system which is impaired with age, and at which time hypertension also develops. The increased intracranial pressure leads to compensatory hypertension via Cushing’s mechanism, i.e., the selfish brain hypothesis. The nasal turbinate vasodilation, due to increased *parasympathetic* activity, occurs simultaneously along with the well-established increased *sympathetic* activity of the cardiovascular system. The increased parasympathetic activity is likely due to an autonomic imbalance secondary to the increase in worldwide consumption of processed food. This hypothesis explains the rapid worldwide rise in essential hypertension in the last 50 years and offers a novel mechanism and a new paradigm for the etiology of essential hypertension. This new paradigm offers compelling evidence for the modulation of parasympathetic nervous system activity as a novel treatment strategy, specifically targeting nasal turbinate regulation, to treat diseases such as hypertension, idiopathic intracranial hypertension, and degenerative brain diseases. The proposed mechanism of essential hypertension presented in this paper is a working hypothesis and confirmatory studies will be needed.

## Summary

Hypertension affects an estimated 1.3 billion people worldwide and is considered the number one contributor to mortality via stroke, heart failure, renal failure, and dementia. Although the physiologic mechanisms leading to the development of essential hypertension are poorly understood, the regulation of cerebral perfusion has been proposed as a primary cause.

Our hypothesis regarding the etiology of hypertension developed from a retrospective review of 200 nuclear medicine scans, where the authors observed a significant increase in nasal turbinate vasodilation in hypertensive patients. The authors propose that the nasal turbinate vasodilation and subsequent increased blood pooling obstruct the flow of cerebrospinal fluid passing through nasal turbinate lymphatics, thereby increasing intracranial pressure. The increased intracranial pressure leads to compensatory arterial hypertension via Cushing’s mechanism. This hypothesis offers a novel mechanism and a new paradigm for the etiology of essential hypertension related to nasal turbinate obstruction of brain lymphatics and suggests possible new treatments for hypertension and degenerative brain diseases. Treating hypertension by methods that focus on nasal turbinate obstruction and/or increasing cerebrospinal fluid lymphatic flow through the nasal turbinates may offer a therapeutic benefit not only to hypertensive patients but to patients with neurodegenerative pathologies as well.

## 1 Introduction

Essential hypertension, also known as primary hypertension, affects an estimated 1.3 billion people worldwide and is considered the number one contributor to mortality via stroke, heart failure, renal failure, and dementia. It is the largest single contributor to global mortality (1). Each year approximately 10 million people worldwide die of hypertension-related disease. The prevalence of essential hypertension is increasing. Between 1990 and 2019, the number of people aged 30–79 years with hypertension doubled from 331 million women and 317 million men in 1990 to 626 million women and 652 million men in 2019 (2, 3). The number of individuals with essential hypertension has steadily increased over the past few decades, likely associated with the large increase in overweight and obese individuals in the world (4, 5). In the United States, the lifetime risk of hypertension surpasses 80% (6). Currently, half of all adults in the United States have hypertension, and the disease is responsible for the highest percentage of all doctor visits (6).

Today nearly 70 percent of what individuals eat in the United States is ultra-processed food. These foodstuffs include packaged chips, energy drinks, and ready-to-heat-and-eat meals. They are thought to be an important driver of the obesity epidemic, in part because they seem to make us eat more (7). This obesity epidemic occurring in the United States has also been noted in other developed and developing countries throughout the world.

Changes in dietary patterns in China, with increased consumption of refined grains and highly processed, high-sugar, and high-fat foods, continue to increase while physical activity levels in all major domains have decreased (5). In China, the number of processed foods available was four times higher in 2013 than in 1999 for a 22.4% annual growth over the 15 years. Over half of the packaged foods sold in China’s markets are processed foods. Overweight, obesity, hypertension, and metabolic syndrome in the Chinese population have become serious public health problems. In 2015, China had the highest number of overweight and obese children globally (5). The increased rate of obesity and hypertension in China likely explains the fact that stroke is now the number one cause of death in that country (8).

Essential hypertension or primary hypertension is not equally distributed in populations worldwide. In the United States, essential hypertension accelerates more rapidly in non-Hispanic Black individuals (NHB) than in non-Hispanic White individuals (NHW) and is often more severe with higher mortality (9). In 2020, age-adjusted hypertension-related NHB adult death rates were approximately twice that of NHW adults (325.3 thousand for NHB men compared with 175.7 thousand for NHW men and 216.1 for NHB women compared with 127.9 for NHW women) (9).

In current medical practice, lifestyle changes are often mentioned as a first line of therapy for patients with hypertension. Alterations or modifications in diet, such as the Dietary Approaches to Stop Hypertension (DASH) (10) are encouraged, as are increased exercise, and restriction of sodium intake. Lowering salt intake moderately reduces blood pressure. An updated systematic review of studies where sodium intake was reduced from 2,200 mg/day to 500 mg/day for 1 week found that the median within-individual change in mean arterial blood pressure between high and low sodium diets was 4 mmHg (11). A newer, more invasive therapeutic technique to control high blood pressure involves renal nerve ablation which reduces sympathetic nervous system activity in the kidney (12).

Today, the most commonly prescribed antihypertensive drugs according to the latest guidelines are combination drug therapies that block the renin-angiotensin system and increase sodium excretion (6). With the rise of renin-angiotensin-targeted drugs, therapies that specifically target only the sympathetic nervous system have significantly decreased in use, even though one of the most verified findings in essential hypertension is that increased sympathetic nervous system activity is associated with the onset of hypertension (12–16). The etiology of this increased sympathetic activity, however, remains controversial as discussed in a recent review of autonomic dysfunction in essential hypertension (13).

Although many different theories about the cause of essential hypertension have been proposed, including excessive salt intake, renal mechanisms, and stress, for most adults there is no clearly identifiable cause with many investigators ascribing the mechanisms of hypertension to multiple factors including interactions between diet and lifestyle, an individual’s gut microbiome (17), neuroimmune modulation (18), and genetic (17) and epigenetic factors (19).

It is well known that hypertension is found in families and that there is a hereditary predisposition to developing hypertension with over 100 single nucleotide polymorphisms associated with the disease (17). *Secondary hypertension*, as differentiated from primary or essential hypertension, has a higher prevalence in children (50% of cases) and young adults less the 30 years of age. Hormonal and primary kidney disease are the main causes of secondary hypertension. Genetic predisposition interacting with environmental influences is a significant contributor to the development of hypertension with the clearest genetic linkages being evident in endocrine hypertension, a form of secondary hypertension (20). Endocrine hypertension has well-defined phenotypes that have allowed patient stratification into homogeneous cohorts. These cohorts can be linked to different genetic variants which have important implications concerning patient therapy (21). Primary aldosteronism is the most frequent form of endocrine hypertension accounting for 5–10% of all hypertensive patients. Several different genetic defects have been linked to primary aldosteronism including autosomal dominant forms and somatic mutations (20).

Although genetic predisposition plays a role in *essential primary hypertension*, the genetic linkages are more complex. In addition, there are significant environmental influences making identification of specific genetic linkages less clear. Although the genetic linkages are less clear, genetic heritability is estimated to account for 40% of blood pressure variance in essential hypertension while environmental influences such as dietary and lifestyle habits can explain the majority of the remaining genetic variance (21). A promising application in the field of hypertension is the use of genetic testing to personalize medical therapy by predicting which anti-hypertensive drugs are most likely to have the greatest effect or cause adverse reactions in an individual patient (22).

Most researchers continue to state that the primary cause of non-endocrine essential hypertension is not well understood (14, 23). This is likely related to the fact that essential hypertension is a multifactorial disease that is considered to be genetically complex with significant interactions with diet and epigenetic factors.

A lack of understanding of the mechanism of essential hypertension contributes to the fact that an estimated 10–30% of patients have resistant hypertension defined as blood pressure that remains above guideline-directed targets despite the use of three anti-hypertensives (including a diuretic) at optimal or maximally tolerated doses (24). Other studies report that the global control rate of blood pressure among people with hypertension was approximately 20% in 2019 (2). A possible explanation for this overall lack of blood pressure control is that the underlying basic pathophysiology leading to the development of essential hypertension is not being addressed. Hence, there is a need to develop new paradigms for understanding essential hypertension with the potential to develop new approaches to therapy.

The objective of this paper is to focus on areas not previously considered as the pathogenesis of hypertension. The authors hypothesize that there is an increase in *parasympathetic* activity in the nasal turbinates that relates to hypertension by causing obstruction of nasal lymphatic drainage, thereby increasing intracranial pressure. The increased intracranial pressure leads to compensatory hypertension via Cushing’s mechanism, also known as the selfish brain. This increased parasympathetic activity of the nasal turbinates occurs simultaneously with the well-established increase in sympathetic nervous activity of the cardiovascular system in hypertension. The increased nasal turbinate vasodilation has been previously described in patients with essential hypertension and other metabolic syndrome features in a recent article by the authors (25). This increased parasympathetic activity results not only in nasal turbinate vasodilation, but also in increased gastrointestinal motility, as observed in hypertensive patients and other patients with metabolic syndrome (26–29).

## 2 Regulation of cerebral perfusion

A less frequently discussed proposed cause of essential hypertension is related to homeostatic processes for the regulation of cerebral perfusion (14, 30). Physiologic processes that impair blood flow to the brain have the potential to lead to increased sympathetic activity and elevated systemic blood pressure to maintain normal blood flow to the brain. The theory has been proposed as the “selfish brain hypothesis of essential hypertension” or “Cushing’s mechanism” (14, 30). Decreased blood flow to the brain and subsequent development of hypertension via the Cushing mechanism has been previously reported to be associated with the narrowing of the vessels supplying the brain (31).

This paper proposes another potential mechanism for the decreased blood flow to the brain that leads to systemic hypertension. It focuses on clinical findings by the authors of increased nasal turbinate vasodilation and resultant nasal blood pooling that causes a restriction of lymphatic flow, or drainage, of cerebrospinal fluid (CSF) from the brain (25). The obstruction of drainage leads to increased intracranial pressure, resulting in increased systemic blood pressure via Cushing’s mechanism (14, 30).

The objective of this paper is to review the literature regarding the above-described physiological mechanisms of hypertension—noting the potential influence of the *parasympathetic* nervous system on increased intracranial pressure—and to propose a novel etiology for this increasingly prevalent disease.

## 3 Increased intracranial pressure and Cushing’s mechanism

### 3.1 Hypertension and increased intracranial pressure

Increased intracranial pressure is present in patients with essential hypertension (32). In 2023, da Costa et al. (32) studied 391 consecutive patients with long-term essential hypertension in an attempt to evaluate intracranial pressure waveforms using a non-invasive device, brain4care. Their study revealed 77.7% of the patients had abnormal measurements of intracranial pressure. The da Costa et al. (32) article was the first to evaluate intracranial pressure behavior in patients with essential hypertension. In addition to their findings, the authors commented that very little is known on the subject of intracranial pressure in patients with hypertension and that they were hoping to “shed some light on the dark side of human history.”

This increased intracranial pressure in hypertensive patients is consistent with the authors’ research showing increased nasal turbinate vasodilation in these same patients. We hypothesize that inappropriately increased nasal turbinate vasodilation with blood pooling in the nasal turbinates is obstructing the normal lymphatic drainage through the nasal turbinates, resulting in increased intracranial pressure.

The authors can find no instances in the medical literature in which invasive lumbar puncture CSF pressure measurements have been performed to study intracranial pressure in patients with essential hypertension. Performing this type of study would be fairly extensive since it is likely the elevations of CSF pressure in many patients would be mild, although significant, in relation to its potential association with essential hypertension.

### 3.2 Cushing’s mechanism

The regulation of cerebral perfusion has been proposed as a cause of essential hypertension with cerebral perfusion pressure being preserved by an increase in systemic blood pressure secondary to increased sympathetic activity (14, 33–36). As early as 1901, Dr. Harvey Cushing proposed the idea of a “Cushing reflex” which he described as a physiological nervous system response to acute elevations of intracranial pressure (ICP) (37, 38). The response consisted of a triad of signs which included widened pulse pressure (increasing systolic, decreasing diastolic), bradycardia, and irregular respirations. He believed that the dramatic increase in blood pressure was a reflex to brainstem ischemia seen in patients with increasing ICP from causes such as intracranial hemorrhage, a mass effect from a tumor, cerebral edema, and other causes. In these studies, Cushing showed that a temporary reduction in cerebral blood flow secondary to increased ICP was associated with a compensatory increase in systemic blood pressure in animals (38). This increase in systemic blood pressure was part of a regulatory process to maintain normal cerebral blood flow.

The human brain is in a tight space, limited by the rigid skull, which makes for a unique situation as it relates to blood and lymphatic flow rates and the strict requirement of the brain to maintain adequate cerebral blood perfusion. Cerebral perfusion pressure is the pressure that pushes the blood through the cerebrovascular network. Cerebral perfusion pressure is a clinical surrogate for the adequacy of cerebral blood perfusion. Cerebral perfusion pressure (CPP) is equal to the mean arterial pressure (MAP) minus the intracranial pressure (ICP) in the following equation (39).


CPP=MAP−ICP

MAP can be estimated as the systolic blood pressure (SBP) plus two times the diastolic blood pressure (DBP) divided by 3.


$$\frac{\mathrm{MAP}=SBP+2X\;DBP}{3}$$

As intracranial pressure increases, the cerebral perfusion pressure (CPP) decreases unless there is a compensatory increase in mean arterial blood pressure (14, 33, 34). With increased ICP, MAP must also increase to maintain adequate blood flow in the brain or CPP. This relationship between ICP and CPP was originally shown by Cushing (37). The authors believe nasal turbinate vasodilatation and subsequent blood pooling obstruct the normal drainage of cerebrospinal fluid from the brain. This obstruction results in increased intracranial pressure (ICP), requiring a compensatory increase in mean arterial pressure (MAP) to maintain cerebral perfusion pressure (CPP). How and where the brain senses its blood flow and is then able to maintain a normal blood flow by increasing systemic blood pressure via the increased sympathetic activity of the heart and vasculature is still a matter of debate.

### 3.3 Cerebral blood flow resistance and hypertension

As early as 1948, Kety et al. (34) reported that patients with essential hypertension had increased cerebral vascular resistance. Their article states that “there is at least some evidence to favor the hypothesis that in essential hypertension there may be a primary cerebrovascular constriction accompanied by a secondary and compensatory hypertension which maintains a normal cerebral blood flow.”

Other researchers (40, 41) confirmed the findings of Kety et al. (34) that cerebrovascular resistance is increased in hypertension and that increased cerebral vascular resistance is the best predictor of the future development of hypertension.

Increased cerebrovascular resistance and increased intracranial pressure have been linked to increased sympathetic activity. In 2018, Schmidt et al. (42) showed that small increases in intracranial pressure would induce a significant increase in sympathetic activity in mice and in humans. In their study in human patients, a 7 mmHg rise in intracranial pressure increased sympathetic muscle activity by 17% as measured by microneurography. This increased sympathetic activity was associated with an elevation in blood pressure.

## 4 Hypertension: a mechanism for self-protection of the brain

Various authors have proposed that hypertension may provide self-protection for the brain by maintaining normal cerebral blood flow as suggested in the selfish brain hypothesis (14, 43).

In 1990, Dickinson (33) wrote an article reappraising the importance of the Cushing reflex for blood pressure stabilization. In this article, he stated that a restriction of blood flow to the brain can produce sustained hypertension. Dickinson also stressed the fact that the Cushing response begins when intracranial pressure begins to rise and is still within the physiological range. He described the Cushing reflex as the most powerful neural blood pressure stabilizing system involving self-protection of the brain.

Paton et al. (30) revisited the idea of self-protection of the brain in 2009, stating specifically that brainstem hypoperfusion could cause the onset of sympathetic hyperactivity and hypertension. They called this the “Cushing’s mechanism,” which was later termed “the selfish brain hypothesis” in an article by Hart (14). Warnert et al. (43) wrote an article that asked the question “Is high blood pressure self-protection for the brain?.” In support of the increase in brain blood flow resistance as a cause of essential hypertension, Hart suggested that congenital vertebral artery hypoplasia is a risk factor for essential hypertension (14). Further studies by her group found that vertebral artery hypoplasia plus an incomplete circle of Willis was associated with lower cerebral blood flow in young adults with hypertension (*p* = 0.0172) (44). This anatomical variant was predictive of hypertension in young adults.

Although this work provides support for the selfish brain hypothesis for subjects with vertebral artery hypoplasia, it would not appear to explain the common and worldwide occurrence of essential hypertension and its near doubling in the number of affected individuals over the last 20 years (2). Instead, the rapidly increasing incidence of hypertension is more consistent with environmental changes likely related to decreased physical activity and diet.

## 5 The autonomic nervous system

### 5.1 The parasympathetic nervous system vs. the sympathetic nervous system

The autonomic nervous system consists of the sympathetic and parasympathetic nervous systems. The sympathetic nervous system controls “flight-or-fight” responses. It prepares the body for strenuous physical activity by increasing the heart rate, elevating blood pressure, heightening awareness, and elevating the respiratory rate. The parasympathetic nervous system carries signals to relax those systems and bring about a state of calm in the body. Parasympathetic responses include an increase of digestive enzymes and more rapid gastric emptying (45), dilation of nasal turbinate blood vessels (46), and decreased heart rate (47).

### 5.2 The paradox of increased sympathetic activity and concurrent increased parasympathetic activity

Perhaps the most verified and agreed upon finding in essential hypertension is increased *sympathetic* nerve activity (14, 36, 48–50). Sympathetic nerve activity, measured by direct microneurography, was found to be increased in hypertension, providing evidence of the involvement of increased sympathetic activity in the development of essential hypertension. Wallin et al. (51) were the first to measure sympathetic nerve activity of the peritoneal nerve and showed that sympathetic nerve activity was increased in hypertensive patients as compared to normotensive patients. Subsequently, the increased sympathetic activity of the cardiovascular system has been confirmed by many investigators (14, 36, 48, 52, 53).

Increased sympathetic activity clearly affects the *cardiovascular system*. How increased sympathetic activity affects *other organ systems* is less well understood, although it has generally been assumed that other organ systems in patients with hypertension experience increased sympathetic activity. As support for the increased sympathetic activity in many organ systems, one article cites a decrease in salivary flow associated with hypertension (54) and suggests that there is a *down-regulation* of *parasympathetic* activity in all organ systems. However, our group reported nuclear imaging-based findings in hypertensive patients that are consistent with *increased parasympathetic activity* in several non-cardiovascular systems (25, 26). Increased *vasodilation* of the nasal turbinates and parotid glands in hypertensive patients, recently reported by our group (25), is consistent with *increased parasympathetic* activity affecting the vasculature of the nasal turbinates, as *increased sympathetic* activity is well known to be associated with nasal turbinate and parotid vascular *constriction*. Abnormally *rapid gastric emptying* in hypertensive patients, as previously reported by our group, is consistent with *increased parasympathetic* activity (26), as increased *sympathetic activity* would have the opposite effect, and *inhibit* gastrointestinal motility. In our study, following ingestion of a liquid carbohydrate meal, hypertensive patients had an average of 41% more rapid gastric emptying compared to non-hypertensive patients (*p* = 0.02), and the rate of gastric emptying correlated significantly with the postprandial glucose level at 30 min (Spearman rank correlation coefficient rs = 0.64, *p* = 0.0428). Our group has also reported that abnormally rapid gastric emptying occurs in spontaneously hypertensive rats (SHR) (55). This rapid gastric emptying observed in humans and in rat models with hypertension is consistent with increased parasympathetic activity of the gastrointestinal system.

As far as the authors know, this paradox of increased sympathetic activity in one region (cardiovascular) while there is simultaneously increased parasympathetic activity in another region has not been previously described (25). This paradox is important for the pathology we will be discussing related to the potential lymphatic obstruction from the brain.

## 6 New glucose set points

The upregulation, or increase in parasympathetic activity, affecting both the gastrointestinal system and nasal turbinate vasodilation that we have observed in our clinical patients, may be related to glucose homeostasis. The authors hypothesize that the increased parasympathetic activity observed in hypertensive patients is due to a resetting of the body’s glucose level. The elevated blood glucose level, or elevated glucose set point, causes a triggering mechanism for an increase in parasympathetically controlled gastric emptying as a means to sustain the elevated glucose levels. Prior studies have shown that an increased gastric emptying rate is an important mechanism for maintaining blood glucose levels (56–58). The increased rate of gastric emptying occurs due to signaling from the hypothalamus via the vagus nerve (59, 60). With a higher glucose set point level, food empties more rapidly from the stomach for absorption into the intestine to elevate and maintain blood glucose levels.

Although the mechanism by which glucose set points become elevated is not clearly understood, the authors hypothesize they become gradually elevated due to the continual increased intake of processed foods. The modern diet, consisting of ultra-processed products, sucrose, and refined grains combined with reduced consumption of fiber, fruits, and vegetables, results in elevated postprandial glucose levels and an upward resetting of the glucose set point. This elevated glucose set point hypothesis is consistent with the significant increase in obesity which has nearly tripled in prevalence since 1960, and the nearly doubling of the number of patients with hypertension over the last 20 years (2).

Our group was the first to report that a gastrointestinal hormone, cholecystokinin (CCK-8), which delays the rate of gastric emptying in patients, had the potential to treat diabetes by lowering postprandial glucose levels (61). We reported that many patients with type 2 diabetes have abnormally accelerated gastric emptying and that infusion of CCK-8 significantly reduced the rate of gastric emptying, which lowered postprandial glucose levels (61). The clinically approved intestinal hormone, glucagon-like peptide 1 (GLP-1), has been widely successful in the treatment of diabetes and obesity. GLP-1 also delays gastric emptying and decreases postprandial glucose levels. Based on the results of our previous studies, GLP-1 would therefore lead to a lowering of the glucose set point. This hypothesis is consistent with the recent findings that GLP-1 agents have been shown to decrease the incidence of cardiovascular disease and stroke in patients with obesity and without diabetes (62). Importantly, GLP-1 drugs have also been shown to result in a modest (5–7 mmHg) lowering of blood pressure that is greater than would be expected from weight loss alone (63–65).

## 7 Nasal turbinate vasodilation and blood pooling

If increased intracranial pressure is indeed a frequent etiology for hypertension, how is it possible, much less probable, that millions of individuals with hypertension have preexisting increased intracranial pressure? The authors believe that increased parasympathetic activity leads to vasodilation of the erectile tissue of the nasal turbinates. These nasal turbinates contain important lymphatic vessels that carry spinal fluid moving through the cribriform plate along the olfactory nerves. We hypothesize that this nasal turbinate vasodilation and blood pooling obstruct the lymphatic cerebrospinal fluid (CSF) drainage leading to increased intracranial pressure.

**Figure 1:**
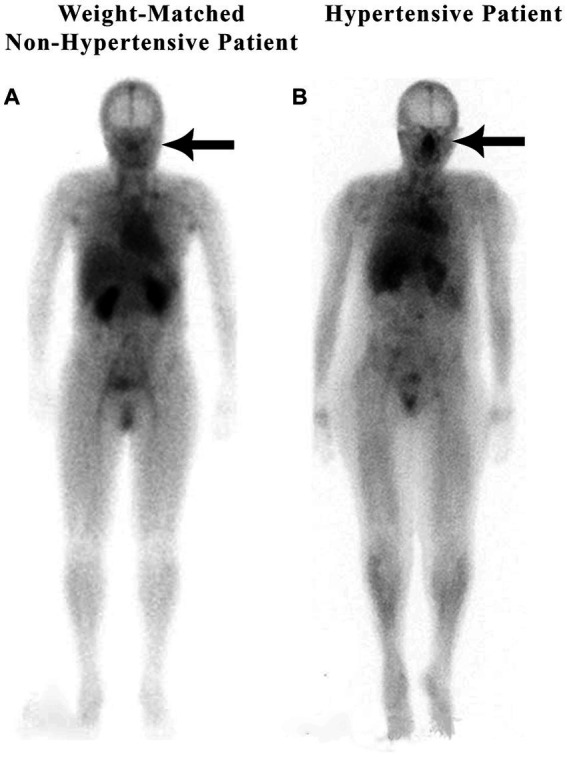
Weight-matched, non-hypertensive **(A)** vs. hypertensive patient **(B)**, both with normal BMIs.

In a retrospective study of 200 patients referred for a routine bone scan, the authors observed that hypertensive patients have significant nasal blood pooling, i.e., increased nasal turbinate vasodilation, as compared to patients without hypertension (25). This increased nasal vasodilation in patients with hypertension is illustrated in Figure 1. The methodology used for obtaining the nuclear scan and the whole-body blood pool imaging is described in the following section.

## 8 Scintigraphic imaging

Scintigraphic imaging of the nasal blood activity in comparison with the cardiac blood activity was determined during the 7-min interval immediately following injection of a bone avid radiopharmaceutical, technetium-99 m methylene diphosphonate (^99m^Tc-MDP), when the radioactivity was in the blood, and before it had time to begin accumulating in the bone. The same scintigraphic imaging technique was used for all 200 patients in this retrospective analysis of whole-body blood pool scanning. Each scan was obtained beginning 2–3 min after injection of the bone avid radiopharmaceutical and took a total of 6–7 min to scan from head to feet. The early images of the bone avid radiopharmaceutical, within the first few minutes after injection, are considered to be markers of the patient’s blood pool, as the radiopharmaceutical requires approximately 3 h for bone deposition and clearance of activity from the soft tissues. Images were obtained with a dual-headed gamma camera (GE Infinia Hawkeye 4, Boston, MA) using low-energy, high-resolution collimators with an energy window set at 140 keV and with a 20% window moving at a rate of 36 cm/min (25). With scintigraphic imaging, it is possible to determine the distribution and activity of blood in the nasal region as compared to the cardiac region.

## 9 Measurement of nose/heart ratios

Nose/heart ratios were determined by placing a square region of interest box over the area of the nose on the nuclear scan. The activity in the maximum pixel was determined in each box, and a ratio of the maximum pixel in the nose was divided by the maximum pixel in the heart. Using the maximum pixel activity is very similar in technique to analyzing the maximum standard uptake value (MaxSUV) as determined in PET imaging for monitoring cancer metabolism. The use of a box and maximum pixel activity decreases the subjectivity incurred with drawing an outline around the whole organ. In our retrospective study of 200 patients, those patients with hypertension had an average nose-to-heart max ratio of 0.93 versus 0.85 in non-hypertensive patients (*p* = 0.0123 using the Wilcoxon rank-sum test) (25). Figure 1 demonstrates a normal-weight non-hypertensive control subject (A) compared to a normal-weight hypertensive patient with increased nasal pooling (B).

## 10 Nasal blood pooling observations on nuclear medicine scan

### 10.1 Increased nasal blood pooling in a normal weight-matched, hypertensive patient vs. a non-hypertensive patient

#### 10.1.1 Increased nasal blood pooling in weight-matched, obese, hypertensive vs. non-hypertensive patients

**Figure 2:**
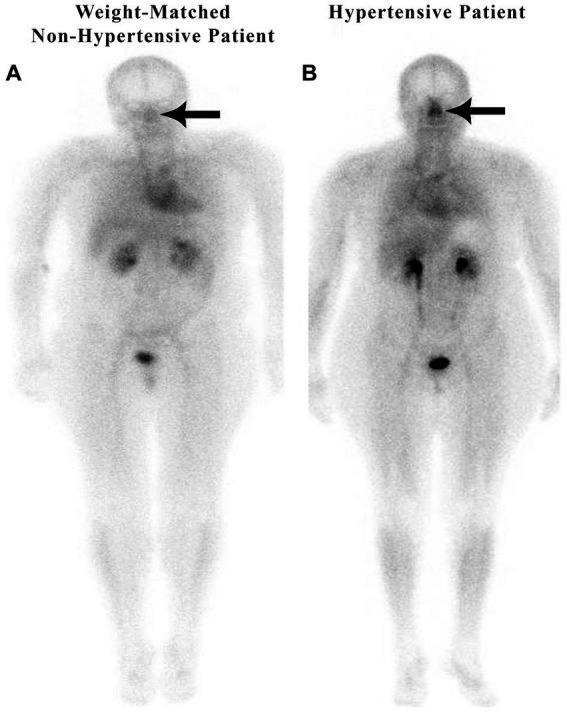
Weight-matched, non-hypertensive **(A)** vs. hypertensive patient **(B)**, both with elevated BMIs.

Figure 2 illustrates the nuclear medicine scan of weight-matched patients, both with an elevated body mass index (BMI). Patient A is a normal control and Patient B has hypertension and hyperlipidemia, but not diabetes or sleep apnea. There is increased nasal blood pooling in the overweight hypertensive patient compared to the overweight non-hypertensive control subject.

Both non-hypertensive patients in Figures 1, 2 have very minimal blood activity in their nasal turbinates while both patients with hypertension have very significant activity in the nasal turbinate region. These whole-body blood pool imaging studies have provided insights to the investigators which have led to their proposal of a working hypothesis described in this paper regarding a new causation paradigm for essential hypertension. Confirmation of these findings will be important. Potential methods to confirm these findings will be addressed in section 19.

### 10.2 Increased blood pooling in erectile tissue of the nasal turbinates

Studies of computed tomography (CT) (66) and magnetic resonance imaging (MRI) (67) have shown that the erectile tissue in the nose is located in (1) the whole of the inferior turbinate (anterior, middle, and posterior), (2) the middle turbinate (more prominent at the middle and posterior turbinate), and (3) the anterior portion of the nasal septum.

The rapidity in which these turbinates can dilate and contract has led two different investigators, Cole et al. (66) and Ng et al. (67), to conclude that nasal turbinate dilation is due to an increase in blood volume in the nasal turbinates and not due to an increase in edema or interstitial fluid. This purported increase in nasal blood pool volume is consistent with our findings of high nasal blood activity in the turbinate region observed on nuclear imaging.

The vasodilation associated with increased nasal turbinate *parasympathetic* activity is the opposite of the vasoconstrictive effect of *sympathetic* activity on the nasal turbinates and the well-known vasoconstrictive effect of sympathomimetic decongestants.

The parasympathetic innervation of the nasal turbinates is delivered through nerve fibers that reach the nasal turbinates through the posterior nasal nerve which crosses the sphenopalatine foramen and distributes to the mucosa following the branches of the sphenopalatine vessels (68). The result is vasodilation of erectile tissue in the nasal turbinates obstructing CSF lymphatic drainage.

### 10.3 Increased nasal blood pooling in patients with metabolic syndrome

**Figure 3:**
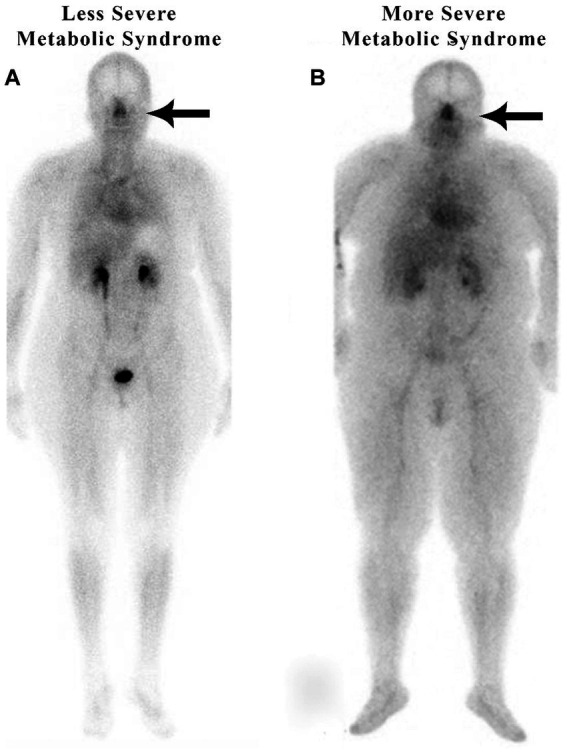
Nuclear medicine scans of patients with metabolic syndrome.

The authors recently published an article that described subjects with metabolic syndrome, including hypertension, increased BMI, diabetes, and sleep apnea, exhibiting significantly increased nasal blood volume (2–3-fold greater), also referred to as blood pooling, as compared with subjects without metabolic syndrome as determined by whole-body nuclear imaging (25). This unique phenomenon of nasal pooling has been observed by the author using scintigraphic whole-body imaging in patients with metabolic syndrome, regardless of their body habitus (Figure 3).

Figure 3 shows increased nasal blood pooling in both patients with metabolic syndrome, including essential hypertension. Patient A, a 59-year-old female with a BMI of 32.5, has less severe metabolic syndrome. She has hypertension and hyperlipidemia with high triglycerides and is being treated with 1 anti-hypertensive and 1 anti-hyperlipidemic medication. Patient B has more severe metabolic syndrome with a BMI of 43.3. The patient is a 55-year-old female with hypertension, diabetes, sleep apnea, and hyperlipidemia, and is being treated with 3 anti-hypertensive, 1 anti-hyperlipidemic, and 1 anti-hyperglycemic medication.

As in patients with hypertension and without complete metabolic syndrome, we hypothesize that those patients with metabolic syndrome have an increase in blood volume in their nasal region significantly decreasing the normal lymphatic transport or drainage through the nasal turbinates resulting in increased intracranial pressure. The increased intracranial pressure causes the increased systemic blood pressure as part of Cushing’s mechanism, i.e., the selfish brain’s attempt to maintain cerebral blood flow. Patient A, the patient on the left in Figure 3, has a less severe form of metabolic syndrome (without diabetes). She, however, has three of the five criteria of metabolic syndrome, including triglycerides over 150, a high waist circumference, and high blood pressure. Nonetheless, she has a high nose/heart ratio.

## 11 Controversy over which comes first, essential hypertension or increased brain blood flow resistance

There continues to be controversy in this area with most researchers believing that it is the development of essential hypertension from a mechanism *not related to the brain* that occurs first and leads to increased resistance to brain blood flow. Far fewer researchers believe that the brain is involved in the initiation of hypertension as proposed by Jennings et al. (69, 70), or that an initial increase in resistance of brain blood flow leads to the development of essential hypertension as proposed by Dickinson and Thomason (71), Paton et al. (30), and Hart (14).

To understand the relationship between decreased nasal drainage, increased intracranial pressure, and hypertension, one must first be familiar with the normal nasal cycle.

## 12 CSF lymphatics and the normal nasal cycle

The nasal cycle is the alternating of airflow between nostrils that shifts between the left and right sides over time (72). The physical mechanism causing the nasal cycle is due to an asymmetry in blood flow leading to the engorgement of erectile tissue in the inferior turbinate and the anterior part of the nasal septum in one nostril more than the other. This normal asymmetrical enlargement of a nasal turbinate on one side blocks the passage of air. The autonomic nervous system mechanism is important in controlling the nasal cycle with sympathetic dominance associated with vasoconstriction and decongestion in one nostril while simultaneous parasympathetic vasodilation and congestion occur in the other nostril (72).

The purpose of the nasal cycle has been debated. Some studies suggest that the nasal cycle is a method of air conditioning and for removing entrapped contaminants (73). Eccles has proposed that the nasal cycle is a mechanism of respiratory defense against infection with respiratory viruses (74). Others have proposed that the nasal cycle could be a way to squeeze interstitial fluid out of the nasal turbinates during the constriction phase of the nasal cycle.

Although it has not been proposed that the nasal cycle serves as a pump to move lymphatic fluid from the CSF into the head and neck lymphatics, the authors believe that this could be one of the most important functions of the nasal cycle.

A malfunction of this normal cycle, with near-permanent vasodilation of the nasal erectile tissue, would result in a blockage of lymphatic outflow from the brain. In this regard, it is interesting that the nasal cycle was found to be diminished with age (75, 76). In one study, 50% of patients over the age of 70 showed no evidence of a nasal cycle (76). Following thorough research, the authors were unable to find any current studies examining the effect of hypertension and metabolic syndrome on the nasal cycle. In our nuclear imaging studies of the blood pool, we did not visualize any asymmetry in the distribution of blood in the region of the nasal turbinates. Patients with hypertension in our whole-body blood pool imaging study, who also had a CT scan of the head, demonstrated symmetrically dilated right and left nasal turbinates without evidence of a nasal cycle (unpublished observation).

It is important to understand how CSF lymphatics are cleared from the brain.

## 13 Clearance of CSF from the brain

There has been considerable controversy regarding the most important pathway of clearance of CSF from the brain. For many years, the most accepted theory was that CSF was absorbed by the arachnoid granulations directly into the venous system. This theory has been significantly challenged over the last 40 years as many investigators have shown the importance of lymphatic clearance of CSF, primarily through the cribriform plate into the nasal region. In addition, a recent study using magnetic resonance imaging (MRI) provided evidence that a portion of the CSF is cleared by the parenchymal venous system (77) with only minimal contribution of the arachnoid granulations in CSF clearance. Further studies are required to provide a better understanding of the contribution of CSF lymphatics, the parenchymal venous system, and arachnoid granulation to overall CSF clearance, however, there has been increasing evidence for the importance of nasal lymphatics in CSF clearance (78–84).

## 14 Evidence for significant clearance of CSF through nasal lymphatics

A major proponent of this idea was Johnston et al. (78, 82, 85) whose work contradicted the most accepted theory that the majority of CSF is cleared by the arachnoid granulations. As pointed out by Johnston and Papaiconomou (79), there has been very limited evidence to support the idea that the arachnoid granulations are the primary site of CSF clearance from the brain; however, there has been significant research supporting clearance of CSF through the cribriform plate into the nasal turbinate region. In one study, Johnston’s group found that 30 min after injection of radiolabeled human serum albumin into the CSF, the tissue that contained the highest activity was the middle nasal turbinate which had approximately 6 times more activity than the blood (82). In another study, Johnston et al. (86) reported that approximately one-half of a protein tracer was transported from the CSF to the blood via extracranial lymphatic vessels. In another study by this group, when CSF transport was blocked through the cribriform plate, resting intracranial pressure doubled from 9.2 cmH_2_O to 18.0 cm H_2_O (87). A recent review of the importance of nasal lymphatics in CSF clearance has been published and is titled, “The brain-nose interface: a potential cerebrospinal fluid clearance site in humans” (80).

Since an original report by Schwalbe (88) in 1869, a large body of work in many different species has indicated a role for lymphatic vessels draining CSF in both cranial and spinal regions. However, only recently published anatomical and quantitative studies have shown that connections between the CSF and the extracranial lymphatic system represent a significant route for CSF drainage (83, 84, 89, 90).

A PET imaging study by de Leon et al. (89) showed tracer activity in the nasal turbinates suggesting CSF movement through the cribriform plate and into the nasal turbinate lymphatics. In a recent study by Zhou et al. (83), 92 patients clearly showed activity in the inferior nasal turbinates following intrathecal infusion of an MRI contrast agent. Another recent 2023 study in rats using high-resolution imaging was strongly supportive of lymphatic movement along olfactory nerves. The study concluded that the olfactory nerve pathway into nasal turbinate lymphatics is the major route of CSF clearance from the brain (90).

In another animal model study, infusion of Ringer’s lactate with blue dye into the cisterna magna to increase the intracranial pressure caused a 3-fold increase in cervical lymph node flow and an increase in blue-colored nasal discharge that appeared 48 min after the beginning of the infusion (91). The nasal discharge increased from negligible, before the cisternal infusion, to 11.4 mL/h following the infusion. These studies support the clearance of CSF in cervical lymphatics and nasal fluid.

Ma et al. (92) found that lymphatic vessels were the major outflow pathway of CSF for both large and small molecular tracers in mice. They also found a significant decline in CSF lymphatic outflow in aged compared to young mice suggesting that the lymphatic system may represent a target for age-associated neurological conditions. In another recent study by Yoon et al. (84), a nasopharyngeal lymphatic plexus was found to be a hub for CSF drainage to the deep cervical lymph nodes. This plexus was suggested as a possible target for the treatment of age-related neurological conditions which are known to be associated with decreased CSF transport to deep cervical lymph nodes.

Meningeal lymphatic vessels located along the dural sinuses have been shown to drain into the cervical lymph nodes (93), and are coupled with, and receive drainage from, the recently described glymphatic system within the brain (94) that was first described by Iliff et al. (95) in 2012 and which will be discussed in the next section.

## 15 The glymphatic/lymphatic system

### 15.1 The glymphatic system

The glymphatic system consists of specialized low-resistance spaces known as Virchow–Robin paravascular spaces that permit CSF inflow deep into the neural parenchyma. A detailed review of this glymphatic system has recently been published by the author (WP) and colleagues (96). The glymphatic system runs in the same direction as blood flow which is propelled by pulsations from the arterial vascular wall. This system can deliver protective molecules, such as melatonin, deep into the brain along the periarterial spaces. It also transports protein waste products, such as amyloid and tau degradation products, from the brain via the paravenous spaces (97). The fluid in the paravenous space eventually moves into the subarachnoid space on the surface of the brain where the fluid and any waste material are absorbed into meningeal lymphatic vessels as reported by Aspelund et al. (98) and Louveau et al. (99) in 2015. This network of meningeal lymphatics serves the same purpose as classical lymphatic drainage and is essential for maintaining neurophysiological homeostasis. The fluid in the meningeal lymphatics is then transported out of the brain and moves into cervical lymphatics. Although the precise anatomic pathway taken by this CSF/lymphatic fluid out of the cranial cavity remains to be clearly defined, the greatest evidence supports its movement along the cranial and spinal nerves, with the olfactory nerve thought to be the most predominant (78, 82). Drainage from these meningeal and cervical lymphatics is relatively fast as tracers injected into the brain or CSF accumulate in the cervical lymph nodes within minutes after injection into the brain or CSF (100). The discovery of this glymphatic/lymphatic clearance system has clearly shown that CSF and interstitial fluid are directionally transported within the CNS.

Interestingly, it has been shown that this glymphatic/lymphatic clearance system is impaired with age at which time hypertension also develops (101). Because the glymphatic/lymphatic system plays a key role in the clearance of amyloid-beta and tau proteins, this system has been suggested to represent a new target to combat neurodegenerative disease (102). There is a recent MRI tracer imaging study supporting this theory which showed that impaired peri-olfactory cerebrospinal fluid clearance through the inferior turbinate was associated with aging, cognitive decline, and decreased sleep quality (83).

### 15.2 Importance of lymphatic nasal drainage for brain fluid homeostasis

Abnormally increased parasympathetic-induced nasal turbinate vasodilation and resultant blood pooling that interferes with the normal nasal cycle would be expected to obstruct lymphatic flow from the brain. In a rat model, nasal turbinate lymphatics were shown to be important for the clearance of CNS fluid when intracranial pressure was artificially increased (85). An increase in intracranial pressure by infusion of plasma into the lateral ventricle resulted in elevated pressure in the deep cervical lymph nodes which receive lymphatic drainage from the nasal turbinates. Very recently reported research in a rat model has also shown that CSF moves through the cribriform plate along the olfactory nerve to join lymphatics in the nasal mucosa which then are carried to a nasopharyngeal lymphatic plexus. CSF then drains to cervical lymph nodes through medial deep cervical lymphatics. These medial deep cervical lymphatics carry a significantly greater volume of CSF as compared to the lateral deep cervical lymphatics (84).

## 16 Increased intracranial pressure consistent with arterial wall thickening

In previous studies, narrowing and thickening of the cervical arteries feeding the brain were cited as evidence of the selfish brain hypothesis of hypertension. The theory is that the vessel narrowing, caused either congenitally or due to atheroma formation, causes an elevation of blood pressure as the brain ensures that it has sufficient blood flow through these narrowed arteries acting via Cushing’s mechanism (14, 30, 31). Vertebral artery thickening has been shown to occur in spontaneously hypertensive rats (SHR) before the development of systemic hypertension (30) supporting the selfish brain theory.

We believe another possible explanation for the thickened vessel walls is that increased intracranial pressure causes a back pressure in the arteries feeding the brain which leads to thickening of the cervical arteries. We hypothesize that lymphatic obstruction of CSF outflow through the nasal turbinates causes increased intracranial pressure and it is this increased intracranial pressure that leads to vessel wall thickening and increased systemic blood pressure as part of the selfish brain hypothesis, i.e., Cushing’s mechanism.

Evidence compatible with increased vascular thickening due to lymphatic obstruction has recently been published in the case of lymphedema of the arms in which brachial arteries feeding the lymphedema arm have significantly greater thickening of the arterial walls compared to the non-lymphedematous arm (103). The significantly increased wall thickness was principally due to increased intima-media thickening resulting in 0.38 mm in the lymphedema arm versus 0.31 mm in the normal arm (*p* = 0.0008).

## 17 Glymphatic/lymphatic obstruction of CSF leads to increased intracranial pressure and hypertension

**Figure 4:**
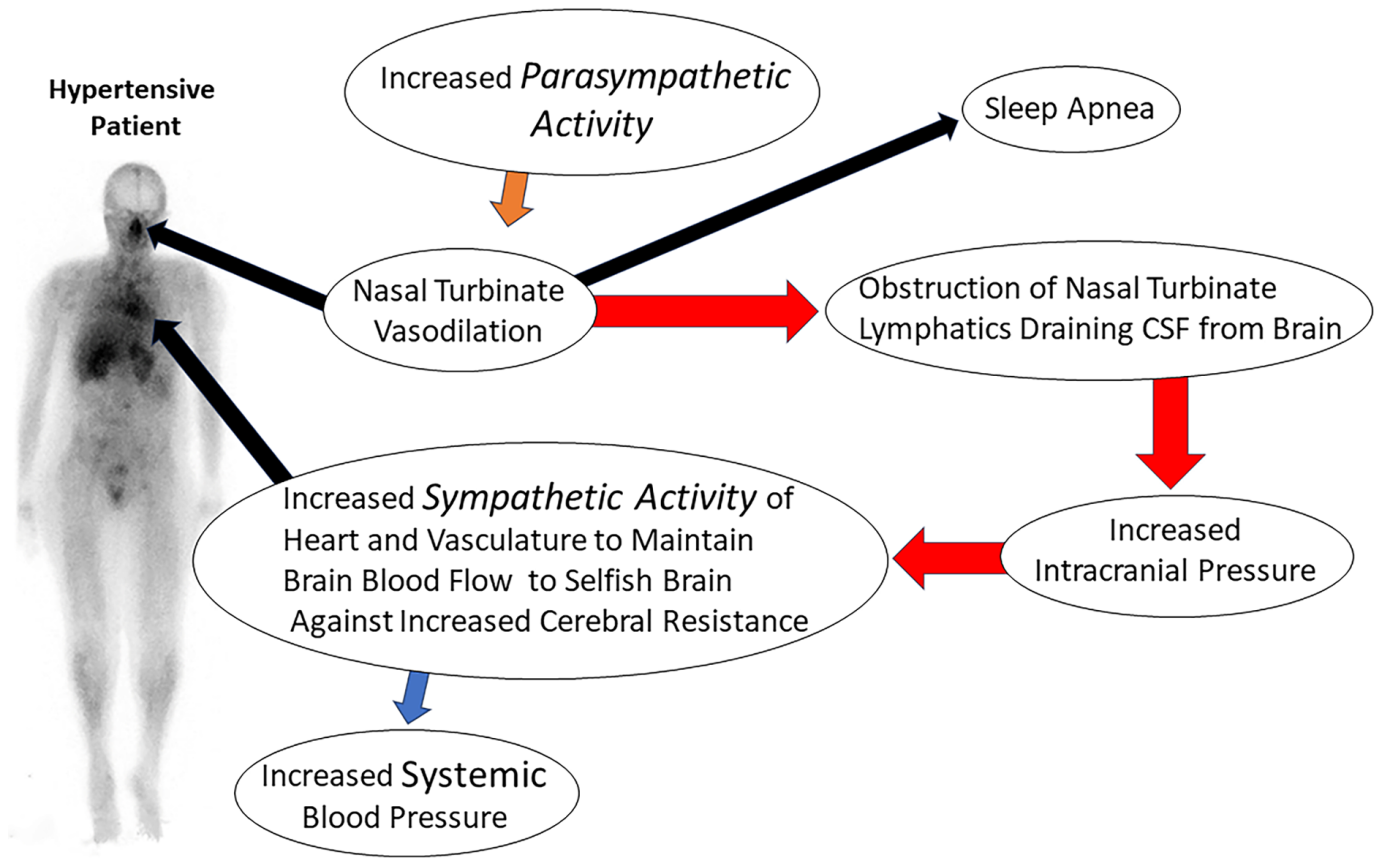
Illustration of consequences of nasal turbinate obstruction leading to increased intracranial pressure and resultant essential hypertension.

The obstruction of CSF lymphatic clearance from the brain at the level of the nasal turbinate due to abnormal turbinate vasodilation and blood pooling would result in decreased clearance of lymphatic fluid from the brain. The decrease in lymphatic fluid drainage would also decrease glymphatic function and the clearance of CSF waste proteins from the brain. Evidence of fluid obstruction in the glymphatic space is provided by MRI imaging in which the perivascular space has been noted to be enlarged in patients with hypertension (104). Evidence of the coupling of the glymphatic system to meningeal lymphatics was first described by Louveau et al. (99) using fluorescent markers. Evidence has also been found for the coupling of these two systems in humans using MRI imaging (94). Prior studies by Johnston (78) and recent PET and MRI imaging studies have shown that significant CSF clearance passes through the nasal turbinates (83, 89). The decreased clearance of fluid from brain lymphatics and the glymphatic system due to nasal turbinate lymphatic obstruction would lead to increased intracranial pressure and a subsequent increase in the systemic blood pressure required to maintain normal blood flow to the brain via Cushing’s mechanism (Figure 4).

Mildly increased intracranial pressure due to lymphatic obstruction would also explain the significant correlation our group observed with increased nasal blood pooling and headaches (unpublished observations to be submitted soon). Similarly, the vasodilation of nasal erectile tissue caused by sildenafil, a drug commonly used to treat erectile dysfunction, causes symptomatic nasal obstruction and headaches (105). Sildenafil is one of the most common causes of drug-induced headaches (106).

## 18 Sleep disturbances

### 18.1 The importance of sleep for adequate CSF lymphatic drainage

Numerous associations have been documented between sleep disturbances and the failure to clear waste products from the brain (107). Sleep disturbances are associated with increased CSF metabolite concentrations (e.g., amyloid-beta, orexin, tau proteins), and increased CSF volumes or pressure (108). Recent studies have suggested that glymphatic dysfunction is a common underlying etiology of sleep disorders and headache pain (109). The glymphatic system is particularly active during sleep whereby potentially toxic neural waste substances that accumulate during wakefulness are cleared via the glymphatic system (108, 110). It is thought that the brain cell volume decreases during sleep, expanding the size of the paravascular space, and facilitating the influx of CSF into the peritubular space for material exchange and metabolic waste removal (111). Animal experiments using intravital 2-photon microscopy in mice showed that glymphatic clearance is decreased by 90% during wakefulness, while protein clearance in the intima of the brain doubles during sleep (97, 112).

Short sleep duration has also been associated with essential hypertension in many epidemiologic studies (113), although there has been no clear pathophysiologic connection found between the two. It is the authors’ hypothesis that decreased CSF clearance due to short sleep and obstructed nasal lymphatics is related to the development of hypertension.

### 18.2 Sleep apnea and hypertension

Obstructive sleep apnea (OSA), another common disorder, is strongly associated with the development of hypertension (114–116) and recent evidence suggests that it may also be linked with cognitive decline and dementia. Sleep apnea may be the common pathway linking hypertension and the development of dementia (117).

**Figure 5:**
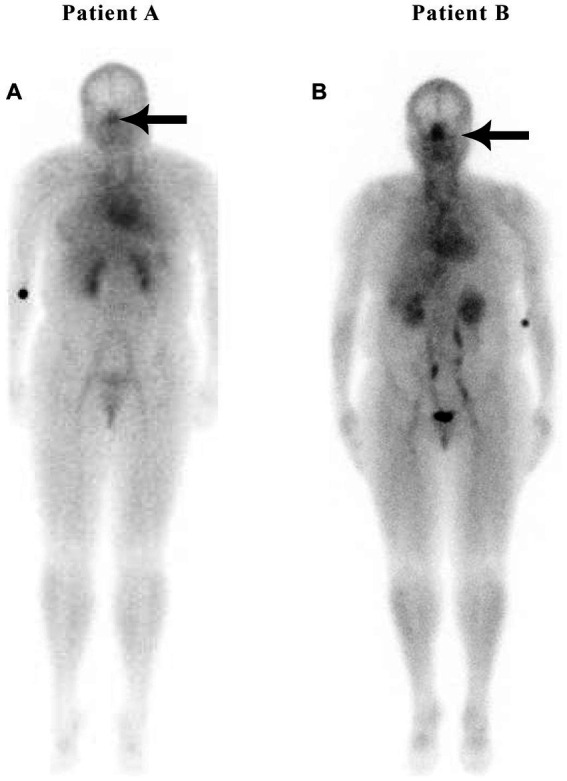
Patients without **(A)** and with **(B)** sleep apnea.

The authors have found significantly increased nasal turbinate blood pooling in patients with OSA. In our review of 200 patients, sleep apnea patients had an average nose-to-heart max ratio of 0.99 versus 0.86 in patients without sleep apnea (*p* = 0.0002) using the Wilcoxon rank-sum test (25). An example image of a patient with sleep apnea is shown in Figure 5. Subjects with OSA have also been shown to have increased sympathetic activity and sleep apnea has been linked to resistant hypertension (118). Although not yet investigated, based on our findings of increased nasal turbinate vasodilation in patients with OSA, it is likely that these sleep apnea patients also have increased nasal parasympathetic activity. In this regard, a recently published Phase 2 study has shown that a drug that reduces the activity of the parasympathetic system significantly improves OSA (119). This drug, taken before bedtime, significantly reduced the apnea-hypopnea index in OSA patients.

A prior study in sleep apnea patients used CT-acquired nasal turbinate measurements to find a significantly positive correlation between the size of the inferior nasal turbinates in obese patients with sleep apnea (120). This prior study did not include patients without sleep apnea so there were no direct comparisons of nasal turbinate size between sleep apnea patients and normal subjects; however, future studies using this CT methodology could be performed to investigate the correlation of nasal turbinate dimensions with hypertension and sleep apnea in the future.

Figure 5 is a nuclear scan of a 49-year-old female (Patient A) without sleep apnea, hypertension, hyperlipidemia, or diabetes, with a nose/heart max ratio of 0.67. Patient B is a 52-year-old female with sleep apnea but without hypertension or diabetes at the time of the study, with a nose/heart max ratio of 1.16. A CT scan at the time of the nuclear study showed dilated nasal turbinates. A 3-year follow-up scan of Patient B showed an increased nose/heart max ratio of 1.28. The patient had developed Stage 1 hypertension and pre-diabetes.

Increased intracranial pressure has been associated with sleep apnea (121, 122), a known risk factor for hypertension (123). Treatment of sleep apnea has been suggested as a method to prevent hypertension (124). The increased intracranial pressure associated with sleep apnea and obesity has even been reported to cause thinning of the skull with an increased likelihood of producing a CSF leak (122). Our findings of nasal vasodilation in patients with hypertension and sleep apnea suggest the possibility that obstruction of the CSF lymphatic clearance from the brain through nasal turbinate lymphatics is responsible for the increased intracranial pressure and the resultant sleep apnea and hypertension. Obstruction of nasal turbinate lymphatic flow as described in this article could also be related to the development of idiopathic intracranial hypertension (IIH). The most common occurrence of IIH is in obese women of childbearing age who are also more likely to have essential hypertension and metabolic syndrome. Sleep apnea has also been associated with both essential hypertension and IIH (116, 125).

## 19 Future confirmatory studies

The proposed mechanism of essential hypertension presented in this paper is a working hypothesis and confirmatory studies will be needed. There is a potential for prospective studies to complement the retrospective studies in patients with hypertension as discussed in this paper. Studies could be performed using nuclear blood pool imaging to assess nasal turbinates as in our retrospective studies. An advantage of the nuclear imaging technique is that dynamic imaging, viewing changes in the nasal blood pool over 8 h, could be performed by simply placing a standard gamma camera over the upper body of the patient. This would allow studies to be performed during sleep or during other medical or physical interventions that affect the nasal turbinates. The gamma camera could be placed several inches away from the patient, resulting in minimal disturbance. To perform prolonged studies, a blood pool imaging agent such as labeled red blood cells could be utilized to permit dynamic imaging for 8–12 h. Technetium-99 m labeled red blood cells are standard blood pool nuclear imaging agents most commonly used for locating the site of gastrointestinal bleeding, diagnosing hepatic hemangiomas, and determining left ventricular ejection fractions (126). Other imaging studies to assess nasal turbinate vasodilation in hypertension could be performed with MRI or CT, such as those previously reported by Rodrigues et al. (120), stating that obese patients had inferior turbinate hypertrophy.

Other studies could be performed with MRI contrast agents, investigating the lymphatic drainage of cerebrospinal fluid through nasal turbinates and its association with hypertension. Studies could also be performed to further assess the absence or presence of the nasal cycle in patients with hypertension as compared to controls.

## 20 Potential novel therapeutic approaches targeting the nasal turbinates

Based on the evidence in this paper, the nasal turbinates are potential targets for the treatment of hypertension. One possible treatment would be to block the increased parasympathetic activity of the nasal turbinates by blocking the sphenopalatine ganglion that carries parasympathetic activity to the nasal turbinates. The sphenopalatine ganglion is the largest extracranial parasympathetic ganglion of the head (127). Sphenopalatine ganglion blockage has been used to treat migraine headaches (127) and a recent study has shown that blocking the sphenopalatine ganglion can modestly lower blood pressure (128). However, completely blocking parasympathetic activity to the nose may not be the best approach for treating hypertension as it would adversely affect the nasal cycle which is dependent on alternating sympathetic and parasympathetic activity to the nasal turbinates (72) which may be important for the clearance of CSF fluid from the brain.

Future therapeutic approaches could be aimed at increasing the volume of CSF flowing through the nasal lymphatics. These therapies’ goal would be to restore the nasal cycle or to use other physical approaches to increase the movement of CSF through and out of the brain. Decreasing intracranial pressure through therapy targeted at the nasal turbinates could lead to significantly improved blood pressure control and a more effective treatment for sleep apnea.

## 21 Conclusion

Finding more effective treatments for essential hypertension offers the possibility of better blood pressure control resulting in a decrease in the incidence of myocardial infarction, stroke, renal failure, dementia, and overall mortality currently associated with hypertension. Considering that accumulation of amyloid and tau proteins in the brain are involved in the pathogenesis of neurodegenerative diseases, the potential of treating hypertension by methods that focus on nasal turbinate obstruction and/or increasing cerebrospinal fluid lymphatic flow may also offer a therapeutic benefit for neurodegenerative pathologies in addition to its potential to treat hypertension.

## Data Availability

The original contributions presented in the study are included in the article/supplementary material, further inquiries can be directed to the corresponding author.
